# Machine learning approaches for estimating interfacial tension between oil/gas and oil/water systems: a performance analysis

**DOI:** 10.1038/s41598-024-51597-4

**Published:** 2024-01-09

**Authors:** Fatemeh Yousefmarzi, Ali Haratian, Javad Mahdavi Kalatehno, Mostafa Keihani Kamal

**Affiliations:** Department of Petroleum Engineering, Amirkabir University of Technology, Tehran, Iran

**Keywords:** Energy science and technology, Engineering

## Abstract

Interfacial tension (IFT) is a key physical property that affects various processes in the oil and gas industry, such as enhanced oil recovery, multiphase flow, and emulsion stability. Accurate prediction of IFT is essential for optimizing these processes and increasing their efficiency. This article compares the performance of six machine learning models, namely Support Vector Regression (SVR), Random Forests (RF), Decision Tree (DT), Gradient Boosting (GB), Catboosting (CB), and XGBoosting (XGB), in predicting IFT between oil/gas and oil/water systems. The models are trained and tested on a dataset that contains various input parameters that influence IFT, such as gas-oil ratio, gas formation volume factor, oil density, etc. The results show that SVR and Catboost models achieve the highest accuracy for oil/gas IFT prediction, with an R-squared value of 0.99, while SVR outperforms Catboost for Oil/Water IFT prediction, with an R-squared value of 0.99. The study demonstrates the potential of machine learning models as a reliable and resilient tool for predicting IFT in the oil and gas industry. The findings of this study can help improve the understanding and optimization of IFT forecasting and facilitate the development of more efficient reservoir management strategies.

## Introduction

Interfacial tension (IFT) is a measure of the force per unit length that acts on the boundary between two immiscible fluids. It is normally measured in dynes/cm^1^. IFT depends on the properties and compositions of the fluids, as well as the temperature and pressure conditions. Understanding the effects of these factors on IFT is crucial for accurately predicting interfacial behavior and optimizing processes in diverse applications. One of the most important applications of IFT is in the oil and gas industry, where it significantly influences diverse aspects, such as enhanced oil recovery, gas injection, pipeline transportation, emulsion stability, acidizing, and carbon capture and storage. IFT between gas–water and oil–water systems is an important parameter in many of these processes, affecting the efficiency, performance, and safety of the operations. For example, in enhanced oil recovery, lowering IFT between injected fluids and crude oil enhances displacement efficiency, resulting in improved oil production^2^. In pipeline transportation, managing IFT is crucial to prevent issues like emulsions and corrosion, ensuring efficient hydrocarbon transportation. Additionally, IFT impacts emulsion stability during processing, affecting separation efficiency^3–5^.

In reservoirs, the gas-oil ratio (GOR) is influenced by IFT, crucial for reservoir management. Furthermore, in carbon capture and storage, understanding and lowering IFT between CO_2_ and brine enhances CO_2_ injection efficiency for secure and long-term storage^6,7^. In acidising operations, the use of IFT reducers such as surfactants can enhance acid penetration and wormhole propagation by reducing the interfacial tension between the acid and the oil or water phase^8,9^.

There are various experimental and theoretical methods to measure and predict IFT for different fluid systems. Theoretical approaches encompass molecular-level theories, such as the Young–Laplace equation and the Gibbs adsorption equation, which relate IFT to interfacial curvature and the distribution of molecules at the interface^10–12^, as well as computational methods, such as density functional theory (DFT) and Monte Carlo methods, which provide insights into the molecular behavior at the interface^13,14^. Experimental techniques involve measuring the force required to deform or detach a fluid interface, such as the capillary rise method, the Wilhelmy plate and Du Noüy ring methods, the spinning drop method, and the pendant and sessile drop techniques, or analyzing the shape and behavior of fluid droplets, such as drop shape analysis and interfacial rheology^15–17^. Despite the progress made in IFT calculations, challenges and limitations persist. Experimental techniques may encounter limitations in terms of accuracy, instrument capabilities, and sample preparation. Theoretical approaches rely on certain assumptions and simplifications, which can affect the accuracy of the calculated IFT. Furthermore, interfacial dynamics and hysteresis effects pose challenges in accurately determining IFT. Looking towards the future, emerging technologies and techniques hold promise for advancing IFT calculations. The integration of nanotechnology, advanced imaging and characterization techniques^18^, computational modeling, and machine learning can further enhance our understanding of interfacial behavior and improve the accuracy of IFT predictions^19^. Machine learning is a branch of artificial intelligence that enables computers to learn from data without explicit programming. Machine learning algorithms can discover complex patterns and relationships in data, as well as make predictions based on new data^20–22^.

In their 2016 research, Mohammad Ali Ahmadi and Behnam Mahmoudi successfully applied the Genetic Algorithm-Least Squares Support Vector Machine (GA-LSSVM) model for estimating gas-oil interfacial tension under reservoir conditions, resulting in R-squared values above 0.9987. Focusing solely on the GA-LSSVM model, the paper demonstrates its excellence in representing the non-linear relationship between variables, with high accuracy and the use of genetic algorithms to optimize model parameters^23^. In 2018, M.P. Andersson and colleagues used a first-principles model and the COSMO-RS implicit solvent model to predict interfacial tension between non-polar oils and water at high temperatures. The study found that interfacial tension decreased significantly for alkanes and aromatic oils above 100 °C, with a linear decline for dodecane and toluene. The study also found a discrepancy in temperature dependence of dodecane-water interfacial tension, suggesting potential measurement issues^24^. In 2019, Menad Nait Amar et al. showcased the Gradient Boosting Decision Tree (GBDT) model as superior in predicting interfacial tension (IFT) for crude oil/brine systems, achieving an R-squared of 0.9977 across all data and outperforming the AdaBoost SVR method. The study enriched the field by developing and statistically validating two machine learning models, with the GBDT model demonstrating high accuracy and utility for IFT estimation^25^. Saeedi Dehaghani and Soleimani (2019) conducted a study to estimate the IFT between CO_2_ and aquifer brine using the SGB model. They used a dataset of 378 experimental data points and found that the SGB model was very accurate and reliable for predicting CO_2_-brine IFT. They also found that pressure was the most important factor affecting IFT. The study showed the advantages of the SGB model over other machine learning models for CO_2_ capture and storage in geological formations^26^. In 2020, Alexsandro Kirch et al. demonstrated the efficacy of machine learning, notably the gradient boosted algorithm, for predicting oil/brine interfacial tensions with an R-squared score of 0.97, overshadowing the less accurate linear regression method^27^. In 2020, Jiyuan Zhang and colleagues conducted a comparative study at China University of Petroleum (East China) to explore the use of machine learning techniques for rapidly estimating the interfacial tension (IFT) between CO_2_ and brine. This research is particularly relevant for CO_2_ injection into underground saline aquifers to combat CO_2_ emissions and address global temperature increases. The study assessed nine machine learning methods and identified extreme gradient boosting (XGBoost) and gradient boosting decision tree (GBDT) as the most robust and capable of providing accurate and fast CO2-brine IFT estimations^28^. In a 2021 study by Menad Nait Amar, the focus was on enhancing genetic programming-based correlations for predicting interfacial tension (IFT) in pure and impure CO_2_-brine systems. Affiliated with Algeria's Sonatrach Département Etudes Thermodynamiques, the research emphasized the significance of accurate IFT predictions for injecting CO_2_ into deep saline aquifers, a critical aspect of carbon capture and sequestration (CCS). This study offers improved genetic programming-based correlations that provide precise IFT predictions across a broad spectrum of operational conditions in CO_2_-brine systems, enhancing their utility for applications related to carbon capture and sequestration ^29^. Zixuan Cui and Huazhou Li (2021) conducted a study to find the best thermodynamic model for CO_2_/H_2_O mixtures. They tested various models and found that the PR EOS model with some modifications was the most accurate for phase compositions and densities. They also proposed a new IFT correlation based on the PR EOS model, which improved consistency. The study showed the effectiveness of their models for CO_2_/H_2_O mixtures ^30^. In their 2021 study, Roy Setiawan and co-researchers assessed various machine learning algorithms to predict the surface tension of binary mixtures containing ionic liquids. The TLBO-ANN model was identified as the most accurate, with the lowest mean squared errors in both training and testing phases. This work advances the computational prediction of surface tensions and highlights the significant potential of machine learning techniques, such as the TLBO-ANN model, in the analysis of ionic liquid mixtures ^31^. Bui et al. (2021) conducted a study to understand the factors affecting water/oil interfacial tension using molecular dynamics simulations. They found that temperature, surfactant density, surfactant tail structure, and surfactant molecular flexibility influenced interfacial tension. They also identified interfacial entropy and enthalpy as key contributors to interfacial tension reduction. The study provided insights for designing better surfactants for various industries ^32^. Yang et al. (2022) studied the interfacial behavior of CO_2_ + H_2_O and hexane + CO_2_ + H_2_O systems with hydrophilic silica using molecular dynamics simulations. They found that the interfacial tension (IFT) and water contact angle of these systems depended on the pressure, temperature, and mole fraction of CO_2_. They also observed that CO_2_ molecules penetrated into the interfacial region between water and silica. Their results have implications for CO_2_-based enhanced oil recovery (EOR) and other geological applications ^33^. Dale Seddon et al.'s 2022 study harnessed the prowess of the XGBRegressor algorithm for predicting complex surface tension profiles of hydrocarbon surfactants, yielding R-squared values up to 0.87. Their approach integrated quantitative structure–property relationships (QSPR) with machine learning, offering a novel framework and contributing valuable computational tools for anticipating surfactant behaviors in solutions ^34^. Nikseresht et al. (2022) presented a novel approach to predict interfacial tension in water/oil systems containing surfactants and salt, highlighting the importance of the Extended UNIQUAC model in describing system behavior and interactions. Their findings shed light on how the presence of certain electrolyte ions and surfactants can influence interfacial tension in these systems ^35^. In a 2022 research article by Mahdaviara et al., the study focuses on assessing the interfacial tension (IFT) in binary systems comprising methane, carbon dioxide, and nitrogen-alkanes. They employ data-driven methods, specifically the Cascaded Feedforward Neural Network (CFNN) and Decision Tree Learning (DT), to model IFT across various gas-alkane combinations. The models consider factors such as pressure, temperature, molecular weight, critical pressure, and critical temperature. The CFNN model outperforms the DT model, displaying higher accuracy with low RMSE values and high R^2^ values in IFT prediction. The study also validates the models through tenfold cross-validation and outlier analysis, highlighting the CFNN model's reliability ^36^. In 2022 , Yingnan Wang et al. employed machine learning algorithms to fine-tune regression equations for gas-alkane binary mixture IFT, achieving exceptional fit with R^2^ values exceeding 0.99 using a cubic polynomial form. Their research advances IFT prediction by comparing the efficacy of the surface excess (SE) model and ML-based equations against traditional parachor models, particularly under high-pressure conditions ^37^. In their 2022 research, Cuthbert Shang Wui Ng and colleagues established that the Multilayer Perceptron-Levenberg Marquardt Algorithm (MLP-LMA) was the superior machine learning model for modeling the interfacial tension (IFT) of the hydrogen-brine system. The study not only demonstrated the highest R-squared value of 0.9998 but also reported consistent preservation of the physical trends in IFT and offered insights into the effects of input parameters on IFT through relevancy factor analysis ^38^. Rashidi-Khaniabadi et al. (2023) conducted a study to model the IFT between surfactants and hydrocarbons for EOR purposes. They used machine learning algorithms, especially GBRT, to predict IFT values based on a dataset of 390 experimental data points and input variables such as temperature, molecular weight, concentration, HLB, and PIT. They found that the GBRT model was very accurate and reliable, and that PIT, concentration, and HLB were the most important factors affecting IFT. The study showed the effectiveness of their models for surfactant-hydrocarbon systems ^39^. In the 2023 study by Afeez Gbadamosi, the potential of hydrogen as a sustainable energy carrier is explored with a focus on underground storage and the critical prediction of hydrogen-brine interfacial tension (IFT) using machine learning. Gbadamosi’s research finds Gaussian Process Regression (GPR) to be notably effective, with the GPR-M2 model outstripping others by 22% in accuracy, although specific metrics such as R-squared are not detailed^40^. In the 2023 research conducted by Johny Mouallem et al., the gradient boosting machine learning method was identified as the most accurate for predicting CO_2_-brine interfacial tension (IFT), achieving an R-squared of 0.964. The study effectively formulated six intelligent models, considering multiple variables, to enhance the forecasting of IFT, which plays a crucial role in CO_2_ geo-storage capacity estimations. Additionally, applying the model to a real UAE carbonate saline aquifer yielded optimal storage depth insights, underlining the practical application of the research^41^.

This paper presents a novel and comprehensive study on modeling IFT of various systems, including oil/gas and oil/water, using machine learning techniques. The results are compared with previous studies that have used different approaches. Table 1 summarizes the main features and findings of the reviewed studies, along with the current study. Table 1 shows that the current study has several advantages and novelties over the existing literature, which are discussed below. The current study considers a wide range of input parameters that affect the IFT of both oil/gas and oil/water systems. These parameters include gas-oil ratio (GOR), oil density, oil formation volume factor (FVF), gas density, gas FVF, and water–gas IFT. Most of the previous studies have neglected some of these parameters or focused on specific cases, such as pure components or binary mixtures. The current study aims to capture the complex behavior of IFT in multicomponent systems by incorporating more input parameters. The current study investigates two types of output parameters, namely oil/gas IFT and oil/water IFT. Among the reviewed studies, only a few have addressed both types of IFT, while most of them have concentrated on one type of fluid pair, such as non-polar oils and water, CO2 and aquifer, hydrogen-brine, surfactants and hydrocarbons, etc. The current study provides a more comprehensive and generalizable model that can be applied to various scenarios and conditions by covering both types of IFT. The current study employs six different machine learning algorithms to predict IFT between gas–water and oil–water systems. These algorithms are Support Vector Regression (SVR), Random Forests (RF), Decision Tree (DT), Gradient Boosting (GB), Catboosting (CB), and XGBoosting (XGB). The performance of these algorithms is compared using various evaluation criteria, such as R-squared, mean absolute error (MAE), root mean square error (RMSE), etc. The current study finds that the Support Vector Regression (SVR) and Catboost (CB) outperform the other algorithms in terms of accuracy and robustness.

**Table 1:** Comparison of current modeling results with previous studies.

Authors (year)	Input models	Output models	Model types	Results	Accuracy
Mohammad Ali Ahmadi and Behnam Mahmoudi (2016)	Pressure Temperature Gas density Oil drop density	Oil/gas IFT	GA-LSSVM	The best machine learning method is the GA-LSSVM	R^2^ = 0.9988
Andersson et al. (2018)	Hydrogen bond temperature dependency exponent Hydrogen bond temperature dependency scale Dispersion temperature dependency exponent Dispersion temperature dependency scale	Interfacial tension between non-polar oils and water at high temperatures	COSMO-RS	COSMO-RS is used for the prediction of the interfacial tension between non-polar oils and water at high temperatures of up to 170 °C	N/A
Menad Nait Amar et al. (2019)	Pressure (P) Temperature (T) Total acid number (TAN) Specific gravity (SG) of crude oil NACL equivalent salinity pH of brine	Crude oil/brine IFT	GBDT, AdaBoost SVR	GBDT model with six inputs was the most accurate	R^2^ = 0.9977 (GBDT) RMSE = 0.7552 (GBDT) RMSE = 1.0867 (AdaBoost SVR)
Saeedi Dehaghani and Soleimani (2019)	Temperature Pressure Concentration of salinity	IFT between CO_2_ and aquifer brine	SGB, RBFNs, MLPNs, LSSVM, ANFIS	The SGB model is more accurate than others	R^2^: 0.99885 for SGB
Alexsandro Kirch et al. (2020)	Oil density Oil composition Salinity concentration	Oil/brine IFT	LR, GBA, DT, RF	GB algorithm had the highest R^2^ score and the lowest errors	R^2^ = 0.97
Jiyuan Zhang et al. (2020)	Pressure Temperature Monovalent Bivalent CH_4_ N_2_	CO_2_-brine interfacial tension	SVM, MLP, KRR, GPR, CART, RF, Adaboost, GBDT, XGBoost	GBDT and XGBoost methods were the most accurate	The R^2^ values for GBDT and XGBoost were higher than 0.98
Menad Nait Amar (2021)	Pressure Temperature Monovalent cation molality Bivalent cation molality The mole fractions of CH_4_ and N_2_	Interfacial tension of the systems pure/impure CO_2_-brine	GP	The best machine learning method in the paper is the genetic programming (GP) approach	R^2^ = 0.9519 RMSE = 3.30
Roy Setiawan et al. (2021)	Specifications of ionic liquids (ILS) and non-ionic liquids Molecular weight of the components Density of the mixture Temperature	Surface tension of binary mixtures containing ionic liquids	TLBO-ANN, PSO-ANN, GA-ANN, CSA-ANN	TLBO-ANN model was the most accurate	MSE = 0.0000002 (TLBO-ANN) MSE = 0.0000004 (PSO-ANN)
Dale Seddon et al. (2022)	Surface tension data of hydrocarbon surfactants from QSPR—molecular descriptors	Complex surface tension profiles of hydrocarbon surfactants	XGBRegressor	XGBRegressor algorithm had the highest R^2^ values and smooth SFT curves	R^2^ = 0.69 (Cmax) R^2^ = 0.79 (log(KL)) R^2^ = 0.87 (log(CMC))
Mahdaviara et al. (2022)	Pressure Temperature Molecular weight Critical pressure Critical temperature	Interfacial tension (IFT) in binary systems comprising methane, carbon dioxide, and nitrogen-alkanes	CFNN and DT	CFNN model is more accurate	AARD:0.9904 (CFNN) AARD:0.9772 (DT))
Yingnan Wang et al. (2022)	Temperature Pressure Alkane molar weight	Gas-alkane binary mixture IFT	ML-based cubic polynomial equations, SE model, paracord model	ML-based equations and SE model outperformed parachor model	R^2^ > 0.99 (ML-based equations), RMSE provided for CO2-alkane mixtures
Cuthbert Shang Wui Ng et al. (2022)	Pressure Temperature Mobility	Hydrogen-brine IFT	MLP-LMA, MLP-Adam, GBR, GP	The best machine learning method is the MLP-LMA model	R^2^ = 0.9998 (MLP-LMA) R^2^ = 0.9990 (MLP-Adam) R^2^ = 1.0000 (GBR) R^2^ = 0.9964 (GP)
Rashidi-Khaniabadi et al. (2023)	Temperature Normal alkane molecular weight Surfactant concentration Hydrophilic–lipophilic balance (HLB) Phase inversion temperature (PIT)	Surfactants and hydrocarbons IFT	DT, ET, GBRT	DT is more accurate	(DT): R^2^ = 0.9939 (ET): R^2^ = 0.9925 (GBRT): R^2^ = 0.9873
Afeez Gbadamosi (2023)	Temperature Pressure Density difference	Hydrogen-brine interfacial tension	GPR, ENN, LR, BTA	GPR-M2 model was the most reliable and accurate	The GPR-M2 model is 22% more accurate than other models
Johny Mouallem et al. (2023)	Pressure Temperature Salinity Salt type CH_4_ fraction	CO_2_-brine interfacial tension	LSBoost, gradient boosting, XGBoost, Gaussian process regression (GPR)	Gradient boosting model was the most accurate and robust	R^2^ = 0.964 RMSE = 2.43
Modeling this article	GOR (gas–oil ratio) Oil density Oil FVF (formation volume factor) Gas density Gas FVF (formation volume factor) Water/gas IFT	Oil/gas IFT Oil/water IFT	(SVR), (RF), (DT), (GB), (CB), (XGB)	Support vector regression (SVR) and Catboost (CB) performed best for Oil/Gas and oil/water IFT prediction	Oil/gas IFT R^2^ = 0.999 (SVR) R^2^ = 0.980 (RF) R^2^ = 0.992 (DT) R^2^ = 0.997 (GB) R^2^ = 0.999 (CB) R^2^ = 0.998 (XGB) Oil/water IFT R^2^ = 0.999 (SVR) R^2^ = 0.980 (RF) R^2^ = 0.873 (DT) R^2^ = 0.938 (GB) R^2^ = 0.986 (CB) R^2^ = 0.977 (XGB)

## Methodology

### Data preparing 

Data preparation is an essential step in the machine learning process, as it affects the quality of the data and the performance of the model^42,43^. Therefore, before applying a machine learning algorithm, data cleaning and preprocessing steps are done to ensure high data quality. Data cleaning involves detecting and dealing with missing values, outliers, and irrelevant or redundant features^42,44,45^. Preprocessing steps involve converting the data into a format that the machine learning algorithm can understand, which may include scaling or normalizing the data to make sure that all features have a similar scale^43^. Data normalization is a technique that transforms the values of a variable or feature into a new range, usually between 0 and 1 or −1 and 1. By scaling down the features, we make sure that they are on a standardized scale, which removes variations in magnitude. This standardization allows for a fair comparison and combination of variables, as they are now on a common scale, enabling accurate analysis and modeling. The normalization process is done by subtracting the minimum value of each index from its actual value, then dividing the result by the range (maximum value minus minimum value) of that index. Normalizing data makes it easier to compare indicators with different units or magnitudes and also helps to speed up the training process^20,42,46,47^. In the exploration of data preparation techniques, the inclusion of tangible examples significantly improves the reader’s understanding of normalization methods. Hence, an example of the Min–Max normalization procedure is now presented:

Imagine a dataset that includes an input feature labeled ‘X’, with its values recorded in units ‘U’, spanning a range from the minimum value ‘X_min’ to the maximum value ‘X_max’. To normalize this particular feature, the Eq. (1) is adhered to:

1 $$\mathrm{Normalized X}= (\mathrm{X }-\mathrm{ X}\_{\text{min}})/({\text{X}}\_\mathrm{max }-\mathrm{ X}\_{\text{min}})$$

Within this equation, ‘X_min’ represents the lowest value observed in the feature ‘X’, while ‘X_max’ denotes the highest value. For instance, a specific value ‘X_value’ from the dataset would undergo normalization as Eq. (2):

2 $$\mathrm{Normalized X}= ({\text{X}}\_\mathrm{value }-\mathrm{ X}\_{\text{min}})/({\text{X}}\_\mathrm{max }-\mathrm{ X}\_{\text{min}})$$

Subsequently, this value of ‘X’ is mapped onto a new scale that ranges from 0 to 1 after the normalization process.By executing this procedure across all numerical features in a dataset, a uniform scale is applied to the data. The process equips machine learning models with the consistency required for improved accuracy and aids in mitigating biases that might distort prediction outcomes.

This section presents a comprehensive overview of the computational techniques employed in the study. Six machine learning methods, namely Support vector regressor, Random Forest, Decision Tree, GradientBoosting, Catboosting and XGBoosting, were applied to computational techniques to develop appropriate models for predicting IFT using Different parameters. The models’ performance was evaluated. A total of 11,075 data were collected, which fully described the crude oil samples. These data were utilized to develop efficient models for predicting IFT more accurately. In the study, the dataset was nearly complete with only a nominal amount of cleaning required to remove a scant number of missing entries. The following stages were meticulously performed on the data to examine the presence of any potential outliers:

- Outlier detection: a thorough statistical analysis was implemented to pinpoint outliers. The Interquartile Range (IQR) method, known for its robustness and its conservative approach to data retention, was utilized. For each quantitative feature, the IQR was calculated. Observations falling below the first quartile minus 1.5 times the IQR, or above the third quartile plus 1.5 times the IQR, were marked as outliers.
- Outlier examination: these identified outliers underwent a detailed assessment. Where outliers were attributed to data entry mistakes or other clear discrepancies, they were omitted from the dataset.
- Review for data integrity: the outliers were scrutinized, especially those potentially reflecting true data variation. This ensured genuine observations, possibly depicting rare but credible scenarios, were preserved. This action was taken to ensure the establishment of predictive models that can accommodate broad data variances, thereby improving their efficacy in practical applications.
- Contextual assessment: experts in the relevant subject matter were consulted to perform a contextual evaluation of each outlier. This measure was essential to guarantee that crucial data were not accidentally discarded from the analytical process.

**Figure 1:**
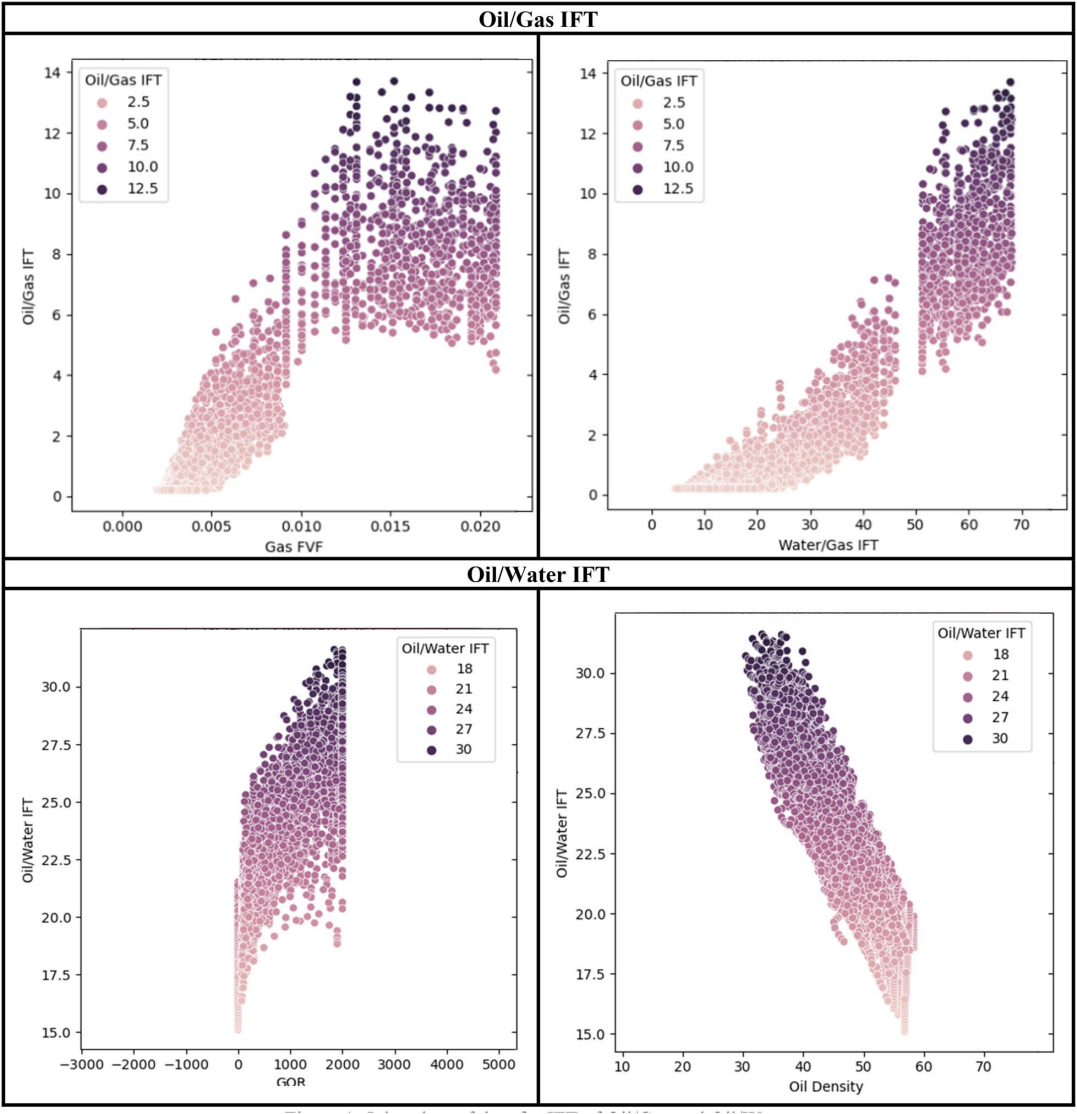
Joint plots of data for IFT of oil/gas and oil/water.

Data joint plots are shown in Fig. 1 to examine the oil/gas IFT and oil/water IFT.

Data used for model development and the IFT range for Oil/Gas and Oil/Water are summarized in Table 2. All input parameters as shown in Table 2, which include GOR (Gas–Oil Ratio), Oil Density, Oil FVF (Formation Volume Factor), Gas Density, Gas FVF (Formation Volume Factor) and Water/Gas IFT, are used in the development of the models for both Oil/Gas and Oil/Water Systems. These parameters were selected based on their significant role in system behavior and their contribution to the accuracy of predictions related to production performance. The experimental databank was randomly divided into two sub-groups: the first sub-group, with 60% of experimental data, trained the models, and the second sub-group, with the remaining 40%, tested the models’ efficiency and reliability against the blind cases. The data allocation method mentioned above often produces desirable and reliable results. In order to check that all the dataset is encompassed, Tables 3 and 4 have been designed to show the statistical ranges after partitioning the dataset. Table 3 presents the minimum, maximum, mean, and standard deviation values of the input parameters for the train and test sets of the Oil/Gas system.

**Table 2:** Statistical ranges and parameters related to inputs/outputs employed for developing models.

No.	Parameter	Unit	Count	Mean	Std	Min	25%	50%	75%	Max
1	GOR	Scf/STB	11,075.0	834.784	622.31	0.100	300.00	700	1300	2000
2	Oil density	lb_m_/ft^3^	11,075.0	45.023	5.676	30.455	40.696	45.085	49.090	58.310
3	Oil FVF	bbl/scf	11,075.0	1.417	0.300	1.000	1.160	1.347	1.632	2.367
4	Gas density	lb_m_/ft^3^	11,075.0	17.678	6.728	2.010	13.895	18.867	22.748	29.463
5	Gas FVF	bbl/scf	11,075.0	0.004	0.003	0.001	0.002	0.002	0.0037	0.020
6	Water/gas IFT	dynes/cm	11,075.0	16.533	15.586	4.585	6.978	9.857	18.120	68.114
7	Oil/gas IFT	dynes/cm	11,075.0	1.138	2.333	0.206	0.206	0.206	0.4613	13.698
8	Oil/water IFT	dynes/cm	11,075.0	23.867	2.861	15.101	21.817	23.696	25.930	31.610

**Table 3:** Statistical ranges and parameters after partitioning the dataset for the train set.

No.	Parameter	Unit	Count	Mean	Std	Min	25%	50%	75%	Max
1	GOR	Scf/STB	6645	844.951	622.220	0.100	300	700	1337.71	2000
2	Oil density	lb_m_/ft^3^	6645	44.973	5.7275	30.7144	40.605	45.025	49.086	58.3104
3	Oil FVF	bbl/scf	6645	1.423	0.3027	1.00	1.1635	1.3558	1.64334	2.3677
4	Gas density	lb_m_/ft^3^	6645	17.714	6.7414	2.011	13.916	18.996	22.7906	29.463
5	Gas FVF	bbl/scf	6645	0.0043	0.00389	0.00196	0.0025	0.00284	0.00374	0.0209
6	Water/gas IFT	dynes/cm	6645	16.5136	15.6208	4.5852	6.949	9.788	18.1397	68.1141
7	Oil/gas IFT	dynes/cm	6645	844.951	622.220	0.100	300	700	1337.71	2000
8	Oil/water IFT	dynes/cm	6645	44.973	5.7275	30.7144	40.605	45.025	49.0863	58.3104

**Table 4:** Statistical ranges and parameters after partitioning the dataset for the test set.

No.	Parameter	Unit	Count	Mean	Std	Min	25%	50%	75%	Max
1	GOR	Scf/STB	4430	819.535	622.213	0.1	292.889	668.849	1300	2000
2	Oil density	lb_m_/ft^3^	4430	45.101	5.59854	30.4552	40.8945	45.1674	49.0967	58.3104
3	Oil FVF	bbl/scf	4430	1.4089	0.2975	1.00	1.155	1.3348	1.6125	2.3036
4	Gas density	lb_m_/ft^3^	4430	17.6255	6.709	2.011	13.8953	18.739	22.631	29.463
5	Gas FVF	bbl/scf	4430	0.004303	0.00398	0.00196	0.0025	0.00283	0.00375	0.0209
6	Water/gas IFT	dynes/cm	4430	16.564	15.5364	4.5852	7.02522	9.944	18.0965	68.1141
7	Oil/gas IFT	dynes/cm	4430	819.535	622.213	0.100	292.889	668.848	1300	2000
8	Oil/water IFT	dynes/cm	4430	45.100	5.59854	30.4552	40.8945	45.1675	49.0967	58.3104

Table 4 shows the same statistics for the Oil/Water system. These tables ensure that both the train and test sets cover the entire range of the dataset and have similar distributions. In addition, 

Table 5 display the hyperparameters’ result as control parameters for each modeling technique used in this study. According to Table 5, among the six models compared for predicting Oil/Water and Oil/Gas IFT, SVR and CatBoostRegressor stand out as the most accurate and reliable models, with the highest R-squared values and the lowest RMSE and MAE values. However, they also have the highest CPU time and memory usage, making them the most costly models in terms of resources. Decision Tree and Gradient Boosting are the opposite, being the most efficient and scalable models, but also the least accurate and reliable. XGBoost and CatBoostRegressor are the middle ground, offering a trade-off between performance and efficiency. Hyperparameters are critical settings that affect the behavior and performance of machine learning models. They are not derived from the data but chosen prior to training and can significantly impact the model’s ability to learn and generalize. Proper hyperparameter tuning is essential for optimizing model accuracy and efficiency. The hyperparameters for each model were tuned using a combination of grid search and cross-validation techniques, with specific focus on improving cross-validated metrics relevant to our study context. The rationale behind the hyperparameters chosen for each model is critical for their optimization and overall effectiveness. Each model’s parameters were carefully adjusted to ensure a robust predictive capability.

**Table 5:** Control parameters used for the development and application of soft computing techniques.

Model	Parameters	Value	Model	Parameters	Value	CPU time	Memory usage
Oil/water IFT			Oil/gas IFT				
Gradient boosting	n-estimators	300	Gradient boosting	n-estimators	300	Seconds to a minute	More memory-efficient than CatBoost and XGBoost
	Max depth	5		Max depth	5		
	Learning rate	0.1		Learning rate	0.1		
XGBoost	n-estimators	100	XGBoost	n-estimators	100	Few seconds to minutes	Similar to CatBoost
	Max depth	7		Max depth	7		
	Learning rate	0.1		Learning rate	0.1		
CatBoost	Depth	8	CatBoost	Depth	6	Seconds to minutes	Few hundred MB to over 1 GB
	Learning rate	0.1		Learning rate	0.1		
	Iterations	300		Iterations	300		
Decision tree	Max depth	5	Decision tree	Max depth	2	Under a second to a few seconds	Very memory efficient
	Max features	None		Max features	None		
	Min samples split	2		Min samples split	2		
Random forest	Max depth	None	Random forest	Max depth	None	Minutes to tens of minutes	Significant, possibly several GB
	Min samples leaf	1		Min samples leaf	1		
	Min samples split	2		Min samples split	2		
	n-estimators	300		n-estimators	300	Minutes to hours	Quite high, especially for non-linear kernels
Support vector regressor	C	100	Support vector regressor	C	100		
	Epsilon	0.01		Epsilon	0.01		

For Gradient Boosting and XGBoost:

- The parameter ‘n_estimators’ specifies the number of trees in the ensemble. This number was empirically determined to achieve a balance between adequate learning from the data and avoiding overfitting.
- The ‘max_depth’ controls the depth of each tree, with a focus on enabling the model to capture complex relationships without fitting to noise.
- The ‘learning_rate’ is utilised to shrink the contribution of each tree, which is pivotal in preventing overfitting and facilitating incremental learning.
- The models were optimized using grid search and cross-validation, with a focus on adjusting n_estimators, max_depth, and learning_rate. This method comprehensively examined a chosen subset of hyperparameters specific to Gradient Boosting and XGBoost.

For CatBoost:

- The ‘depth’ parameter impacts the model’s complexity and guards against overfitting, as CatBoost constructs symmetric trees.
- The ‘learning_rate’ serves to prevent overfitting, similar to its use in Gradient Boosting and XGBoost.
- The ‘iterations’ dictate the number of trees built, similar to ‘n_estimators’, to avoid overfitting while ensuring generalization.
- The CatBoost model’s depth, learning_rate, and iterations were fine-tuned using randomized search. This method assesses a set number of hyperparameter combinations from specific distributions, thus boosting computational efficiency. Cross-validation was employed to ensure thorough evaluation of each parameter set.

For the Decision Tree:

- ‘Max depth’ limits tree complexity to avoid overfitting.
- ‘Max features’ decides the number of features to consider for the best split, adding a level of randomness to each decision.
- ‘Min samples split’ sets the minimum number of samples for node splitting, impacting tree depth and complexity.
- The Decision Tree model underwent grid search optimization similar to the Gradient Boosting and XGBoost models, targeting max_depth, max_features, and min_samples_split adjustments. The best hyperparameters were determined by methodically analyzing combinations in conjunction with cross-validated performance metrics.

For the Random Forest:

- Parameters like ‘Max depth’, ‘Min samples leaf’, and ‘Min samples split’ are used as in Decision Trees, optimizing the balance between bias and variance.
- ‘n_estimators’ defines the robustness of the model, with a greater number of trees potentially leading to better performance.
- The Random Forest model underwent optimization for hyperparameters including max_depth, min_samples_leaf, min_samples_split, and n_estimators through a grid search with cross-validation. A variety of values for each hyperparameter were examined to guarantee an extensive search space.

For Support Vector Regression (SVR):

- The ‘C’ parameter represents the error term’s penalty, trading off decision boundary smoothness against correct classification of training points.
- ‘epsilon’ outlines the epsilon-tube where predictions within a certain range of actual values are not penalized, effectively setting a margin of tolerance for errors.
- The SVR model was refined using a grid search focused on optimizing the C and epsilon hyperparameters. This search aimed to identify the optimal parameter combination that minimizes the generalization error.

### Models

In this study, machine learning algorithms such as Support Vector Regression (SVR), Random Forests (RF), Decision Tree (DT), Gradient Boosting (GB), Catboosting (CB), and XGBoosting (XGB) were employed to calculate the surface tension between water, oil, and gas oil fluids. The selection of these six machine learning models was indeed a critical step in the research methodology and was informed by Selection Criteria:

- Established efficacy: each model was chosen based on its documented success in literature for regression tasks, particularly in fields related to petrochemical studies.
- Variety in learning approaches: we aimed to encompass a diverse set of learning algorithms (e.g., tree-based, ensemble methods, boosting methods) to compare different kinds of learning strategies and their effectiveness in IFT prediction.
- Optimization capabilities: models like XGBoost, Gradient Boosting, and Catboost were included specifically for their advanced optimization techniques that are known to enhance performance with complex datasets.
- Interpretability vs. performance: the selection spans from simple to complex models to evaluate the trade-offs between model interpretability (e.g., Decision Tree) and predictive performance (e.g., XGBoost).
- Relevance to the dataset: considering the nature of our dataset, the selected models are well-suited for handling the types of features and relationships present in the data collected for IFT predictions.

#### Support vector regression (SVR)

SVR is a machine learning method that aims to find a function that approximates the relationship between input variables and a continuous target variable while minimizing prediction errors^48,49^. Unlike Support Vector Machines (SVMs), which are mainly used for classification tasks, SVR focuses on finding a hyperplane that fits data points closely, allowing some deviation^50–52^. SVR was chosen for its established efficacy in handling nonlinear relationships between independent and dependent variables, thanks to its use of kernel functions (such as linear, polynomial, radial basis function, sigmoid), and is particularly favored for its robustness to outliers and solid theoretical underpinnings, evidenced by its reliance on a subset of data points known as support vectors which supports its capability in forecasting continuous outcomes like IFT^53–55^. SVR has been used for IFT prediction under different conditions, demonstrating its adaptability and utility in this field^35^.

#### Decision tree (DT)

DT is a machine learning algorithm that constructs a tree-like structure to represent data and decision rules. DT can be used for both classification and regression tasks. DT employs recursive partitioning, dividing data into subsets based on criteria like information gain or Gini index, creating nodes and final predictions at leaf nodes^56^. DT can handle categorical and numerical features, missing values, and outliers. DT offers interpretability, providing a visual representation of the decision process and requiring minimal data preprocessing and parameter tuning^55,57^. DT excels in capturing complex, nonlinear relationships and feature interactions in data. DT has been applied for IFT regression problems in various studies.

#### Random forests (RF)

RF is a machine learning algorithm that combines multiple decision trees to produce a single result. RF can handle both classification and regression problems^50^. RF employs bagging, a technique that reduces estimator variance by averaging predictions from bootstrap samples, and it also introduces randomness in feature selection at each tree split to enhance diversity and reduce tree correlation^58^. RF has several advantages, such as the ability to handle complex and high-dimensional data, accommodate missing values and outliers, and provide insights into variable importance for predictions. Importantly, RF is resilient against overfitting, as it averages predictions from multiple trees that avoid memorizing noise in the data^54,59–61^. RF has been employed for various regression tasks, such as modeling IFT a crucial physical property used in industries like oil recovery.

#### Gradient boosting (GB)

GB is a machine learning technique that improves the accuracy of predictions by combining simple models into a better, more accurate one. GB works by adding new models that fix errors from previous ones, guided by a loss function that measures how well the model fits the data. GB can use different types of simple models, such as decision trees or linear regressors. GB has several advantages, such as the ability to handle different types of loss functions, optimize both bias and variance trade-off, and prevent overfitting by using regularization techniques. GB has been applied in scenarios where accurate predictions are crucial. For example, researchers used GB with decision trees as the simple models to predict IFT in surfactant-hydrocarbon systems. They used data from previous studies and some extra features. The best results came from Gradient Boosted Regression Trees, with a very small error^56,62^.

#### Extreme gradient boosting (XGBoost) 

XGBoost is an advanced version of gradient boosting that incorporates several improvements and enhancements to the technique. XGBoost uses a second-order Taylor expansion to approximate the objective function, instead of using a first-order approximation as in traditional gradient boosting. This allows XGBoost to capture more complex and nonlinear relationships between input and output variables, as well as handle different types of loss functions. XGBoost also adds a regularization term to the objective function, which penalizes the complexity of the model and prevents overfitting. The regularization term can be controlled by two hyperparameters: alpha (L1 regularization) and lambda (L2 regularization). Moreover, XGBoost employs a column sampling method, similar to random forests, which randomly selects a subset of features at each tree split. This reduces correlation among trees and improves diversity and robustness. Furthermore, XGBoost implements an efficient parallel and distributed computing algorithm, which speeds up the training process and enables scalability to large-scale data sets. XGBoost also includes features like early stopping to prevent overfitting and parameter tuning options for fine-tuning the model. XGBoost is widely used in various machine learning competitions and has become a popular choice among data scientists and practitioners^60,61,63,64^.

#### CatBoost

CatBoost is another gradient boosting algorithm that is specifically designed to handle categorical features effectively. Categorical features are those that have a finite number of possible values, such as gender, color, or country. Categorical features are common in many real-world data sets, but they pose challenges for machine learning algorithms, as they require encoding or preprocessing before being fed to the model. CatBoost provides built-in handling of categorical variables, removing the need for explicit encoding or preprocessing. CatBoost uses a variant of gradient boosting that incorporates a novel algorithm for handling categorical features called Ordered Boosting. This algorithm exploits the natural ordering of categorical variables to improve the gradient boosting process. Ordered Boosting works by randomly permuting the order of the observations in each iteration and using only the observations before the current one to calculate the target statistics for categorical features. This reduces overfitting and leakage of information from future observations. CatBoost also includes features like automatic parameter tuning, built-in cross-validation, and support for GPU acceleration. It is particularly useful when working with data sets that contain a large number of categorical features^47,65,66^.

## Results and discussion

**Figure 2:**
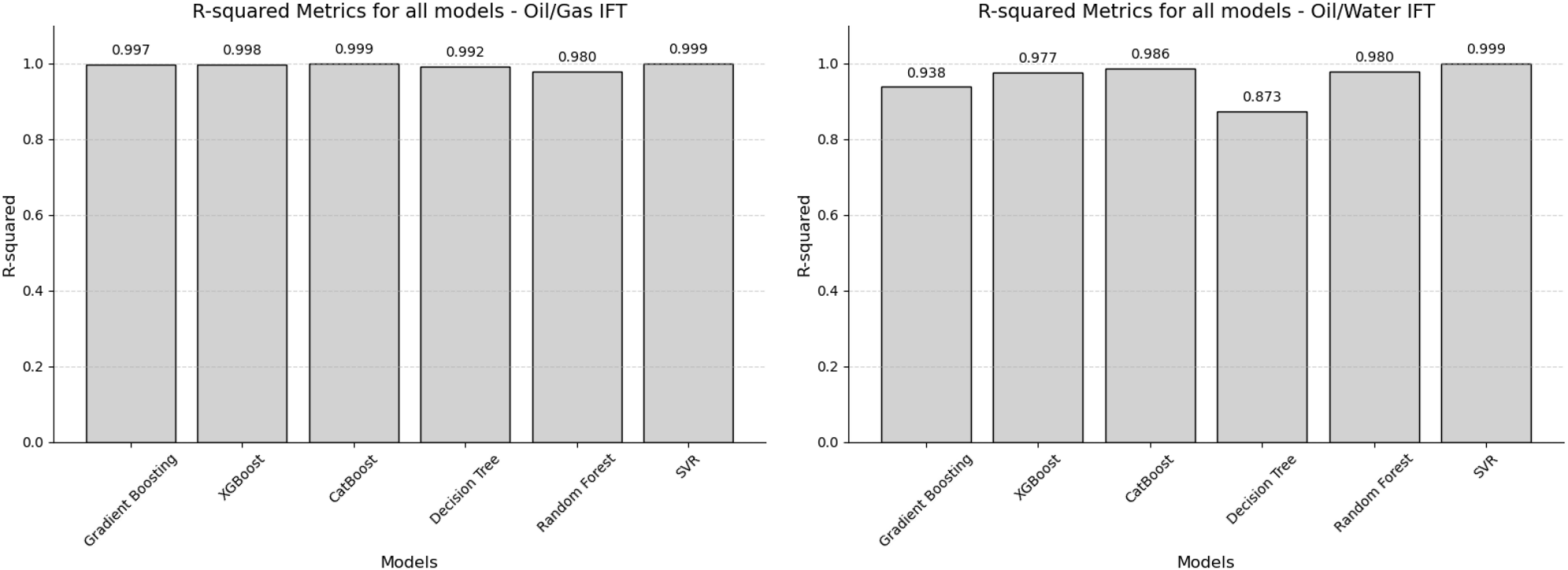
R-squared metrics for all machine learning techniques.

This section presents a comparative analysis of the performance of six machine learning models in predicting the interfacial tension (IFT) between oil/gas and oil/water. These models were introduced in the previous section. Figure 2 illustrates the results obtained from these models. R-squared, or coefficient of determination, is a statistical measure that indicates the proportion of variance in a regression model explained by independent variables, indicating the model's goodness of fit. The R-squared value ranges from 0 to 1, where a 0 indicates that the model does not explain any of the variability in the response data around its mean, and a 1 indicates that the model explains all the variability in the response data around its mean. In practice, an R-squared value of 0.7 or above is often considered acceptable, suggesting that the model has a sufficiently strong explanatory power.

Regarding the prediction of Oil/Gas IFT, Catboost and SVR models achieved the highest accuracy, with an R-squared value of 0.99. Conversely, Random Forest exhibited the lowest accuracy, with an R-squared value of 0.98. In the case of Oil/Water IFT prediction, SVR outperformed Catboost, attaining a higher accuracy of 0.99 compared to Catboost’s accuracy of 0.986. The Decision Tree model demonstrated the lowest R-squared value of 0.873. In the study, R-squared values remained above 0.9, suggesting that over 90% of the variability in IFT could be predicted from the models, which is a strong indicator of excellent model performance given the complexity of the phenomena being modeled. On the other hand, models like Support Vector Regression and linear models can be sensitive to outliers, and their performance depends heavily on the choice of kernel and regularization techn. Additionally, ensemble methods like Random Forest and Gradient Boosting involve multiple decision trees to improve prediction accuracy and handle variance effectively, making them generally more reliable and accurate. Contrastingly, a single decision tree, while interpretable and simple, is often prone to overfitting and may not capture the overall complexity of the data as effectively as ensemble methods.

**Figure 3:**
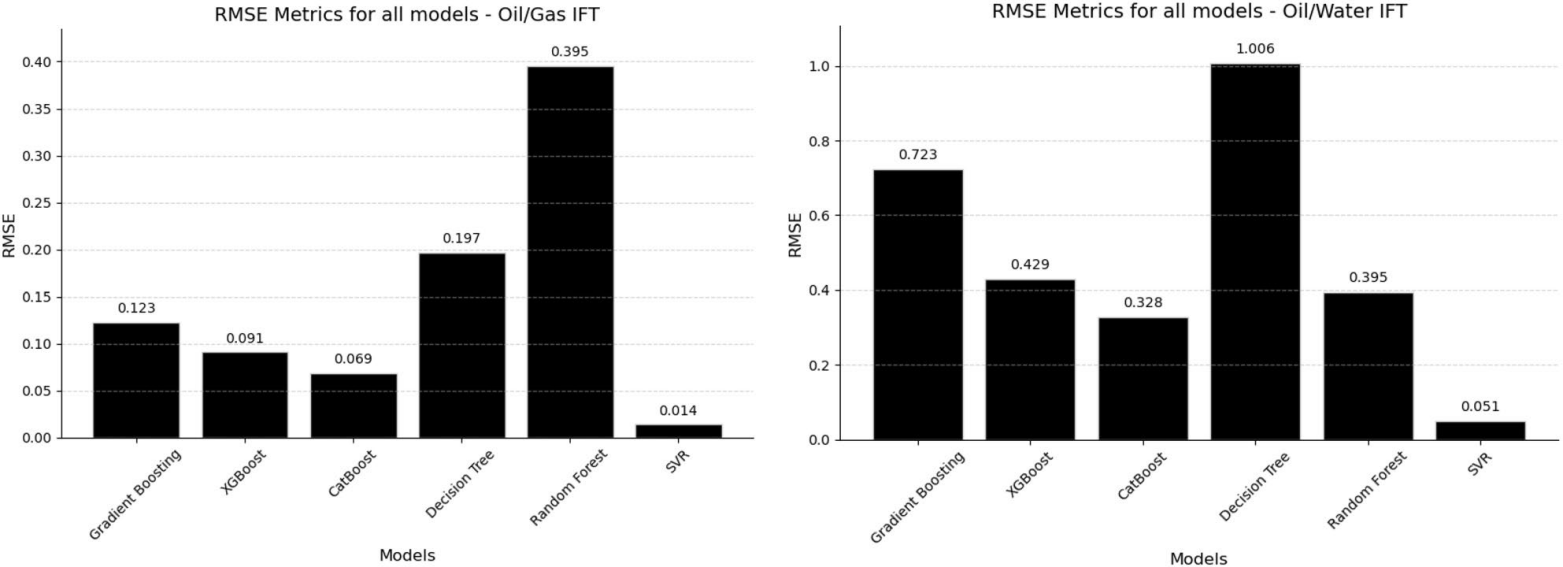
RMSE metrics for all machine learning techniques.

**Figure 4:**
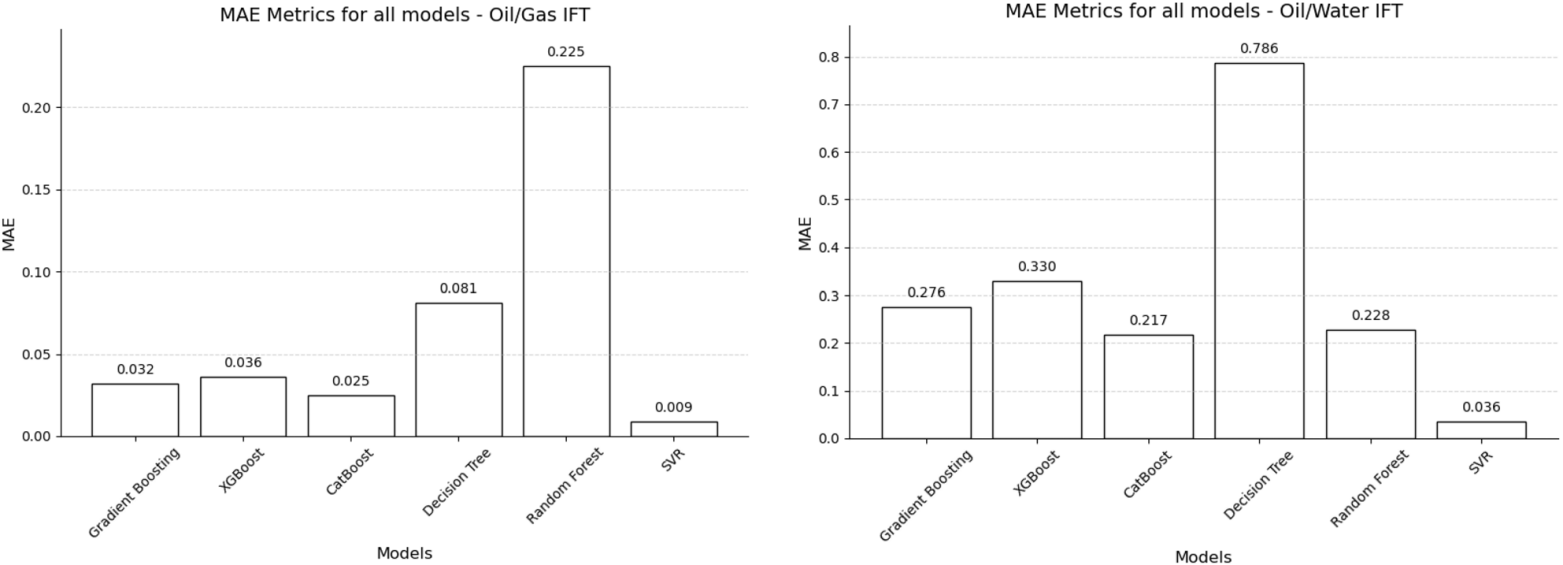
MAE metrics for all machine learning techniques.

Additional evaluation metrics, namely Root Mean Square Error (RMSE) and Mean Absolute Error (MAE), were employed to assess the performance of the models. RMSE measures the average magnitude of errors with a bias towards large errors, while MAE gives the average error size, treating all errors equally. The corresponding results are illustrated in Figs. 3 and 4.

In evaluating predictive models, mean absolute error (MAE) and root mean square error (RMSE) serve as critical metrics. Both gauge the average magnitude of errors by comparing predicted values against actual outcomes. A model’s performance is deemed proficient when it has low MAE and RMSE values, indicating high accuracy and minimal error margins. Conversely, elevated MAE and RMSE values signal deficient performance, characterized by low accuracy and substantial errors. In the context of Oil/Gas IFT prediction, SVR achieved the lowest MAE and RMSE values, measuring 0.009 and 0.014, respectively. Conversely, Random Forest exhibited the highest values for both metrics. Similar to the Oil/Gas IFT scenario, SVR also demonstrated the lowest MAE and RMSE values for Oil/Water IFT prediction. Specifically, SVR achieved a MAE of 0.036 and an RMSE of 0.051. The depicted plots in Figs. 3 and 4 provide a visual representation of these results, highlighting the superior performance of SVR in terms of MAE and RMSE values for both Oil/Gas IFT and Oil/Water IFT prediction tasks. Given the specifics of the dataset, the range for Oil/Gas Interfacial Tension (IFT) is from 0.206 to 13.698 units, and for Oil/Water IFT, it is between 21.817 and 31.610 units. The diversity in scale between these two sets of measurements necessitates a careful interpretation of the metrics. The ranges of MAE in the research are from 0.03 to 0.7 in the context of Oil/Water IFT and 0.03 to 0.2 in Oil/Gas IFT. RMSE is respectively from 0.01 to 0.3 and from 0.05 to 1. The RMSE values obtained were also low in the context of the IFT values, which means that the standard deviation of prediction errors was small, implying high prediction accuracy. The MAE for the models was notably low relative to the range of IFT values, indicating that the average magnitude of the errors in predictions was minimal. An MAE of less than 10% of the range of the output variable is often considered good.

**Figure 5:**
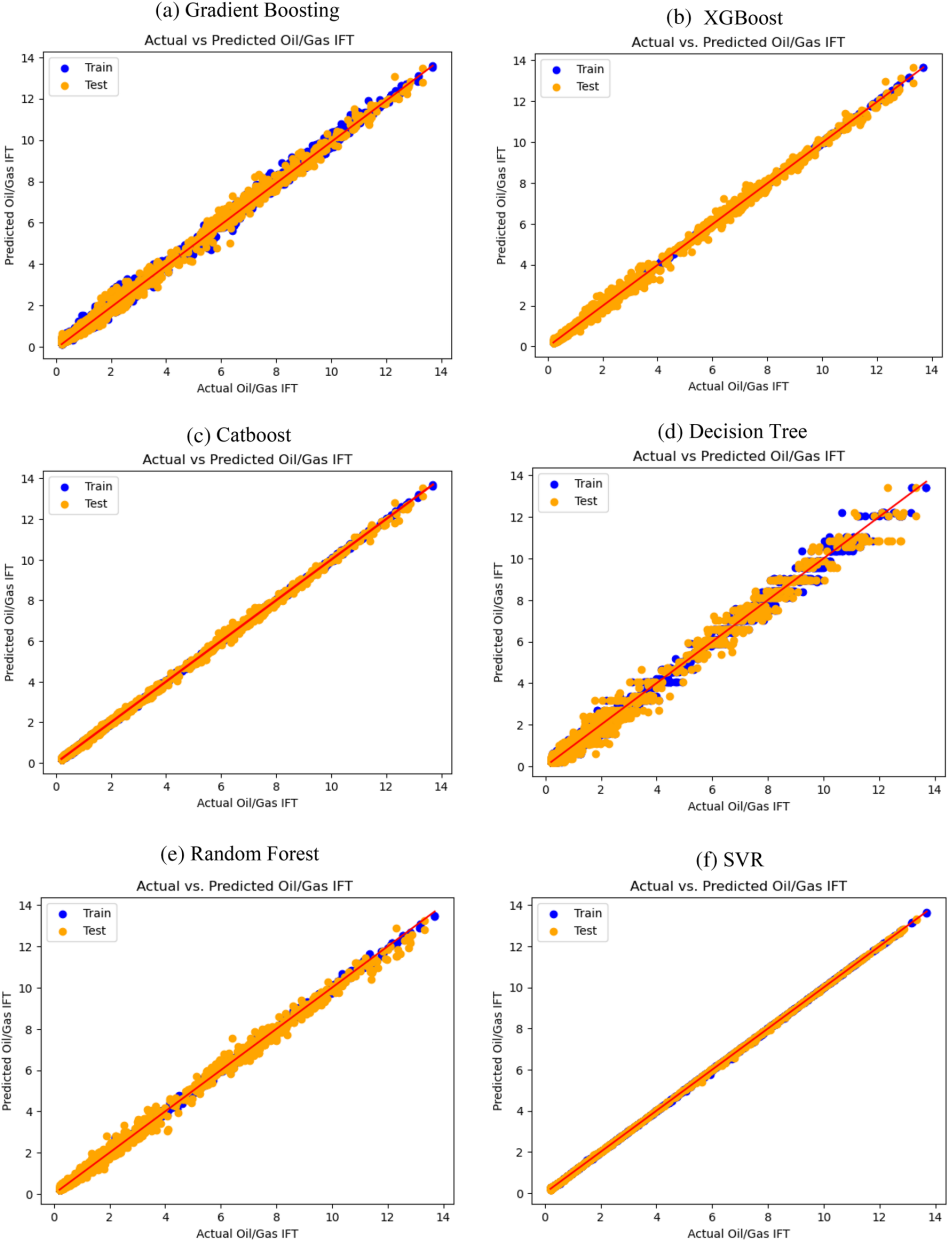
The cross plot of modeling prediction of oil/gas IFT versus measured data. For (**a**) Gradient boosting, (**b**) XGBoost, (**c**) Catboost, (**d**) Decision tree, (**e**) Random forest and (**f**) SVR.

**Figure 6:**
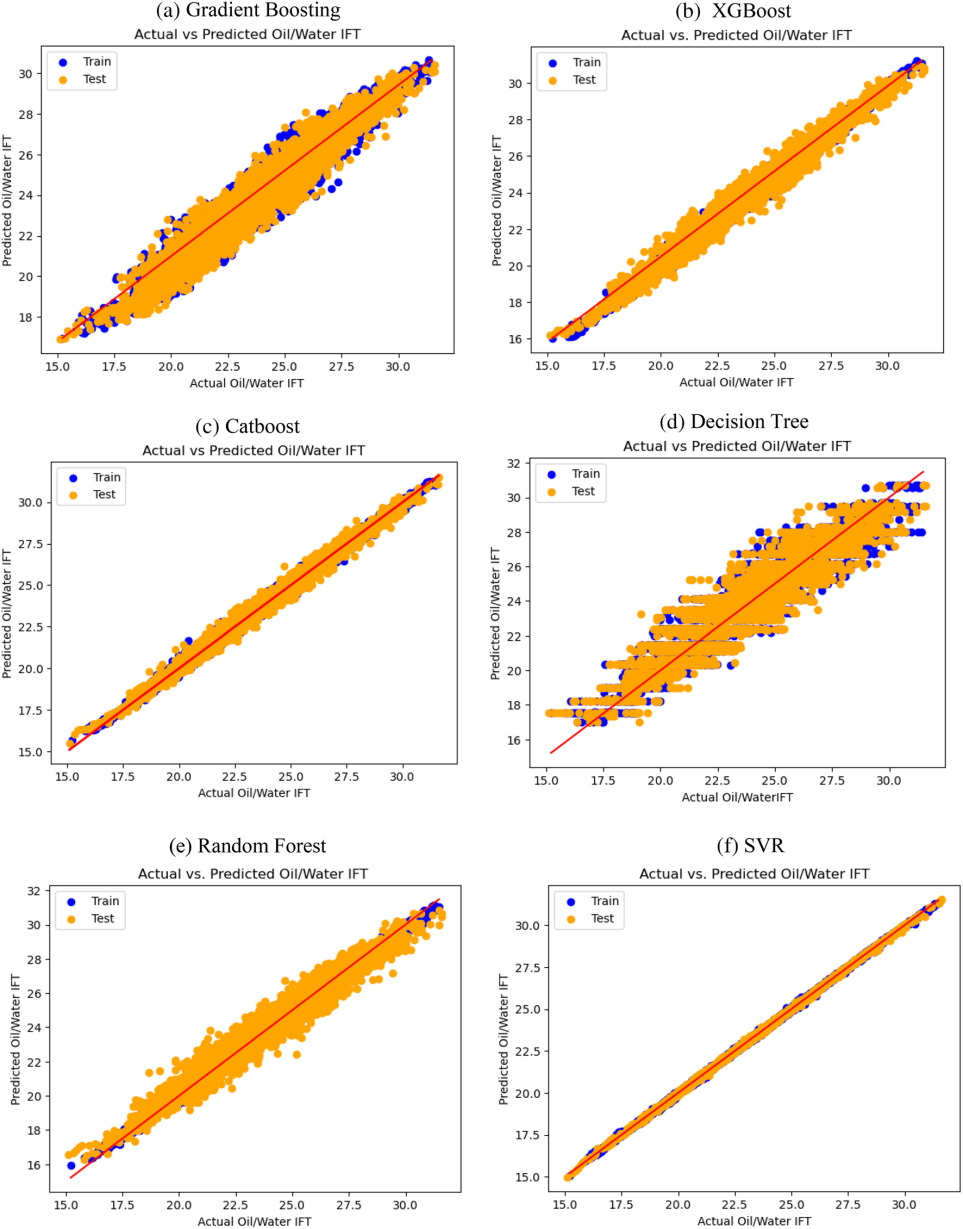
The cross plot of modeling prediction of oil/water IFT versus measured data. For (**a**) Gradient boosting, (**b**) XGBoost, (**c**) Catboost, (**d**) Decision tree, (**e**) Random forest and (**f**) SVR.

Figures 5 and 6 depict the graphical representation of the comparison between the actual data and the predicted data for the specific portion of the dataset where the utilized methods exhibit their optimal performance. These figures offer a visual perspective on the prediction accuracy of Oil/Gas IFT and Oil/Water IFT individually. The generated plots exhibit a comparison between the measured values (horizontal axis) and the predicted values (vertical axis) accompanied by their corresponding regression plot. The dataset has been partitioned into test data and train data, represented by orange and blue markers, respectively.

Analyzing the plots pertaining to Oil/Gas IFT, it is evident that SVR and Catboost models demonstrate the closest alignment between the predicted and measured data. Similarly, for Oil/Water IFT, SVR and Catboost models exhibit the highest level of agreement between the predicted and actual values.

**Figure 7:**
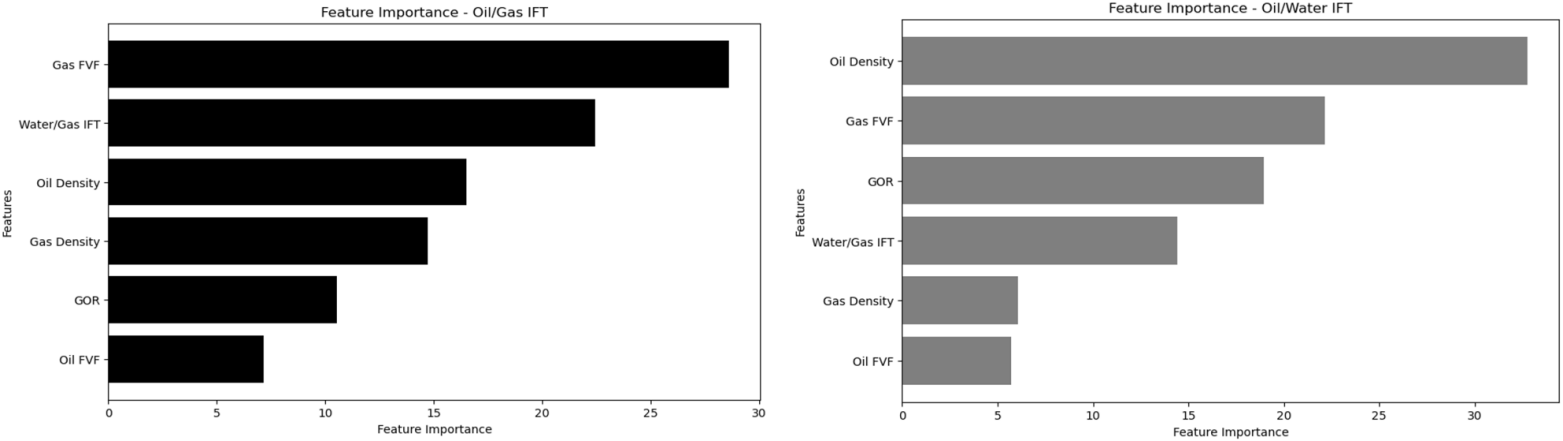
The feature importance of Catboost model for both oil/gas IFT and oil/water IFT.

**Figure 8:**
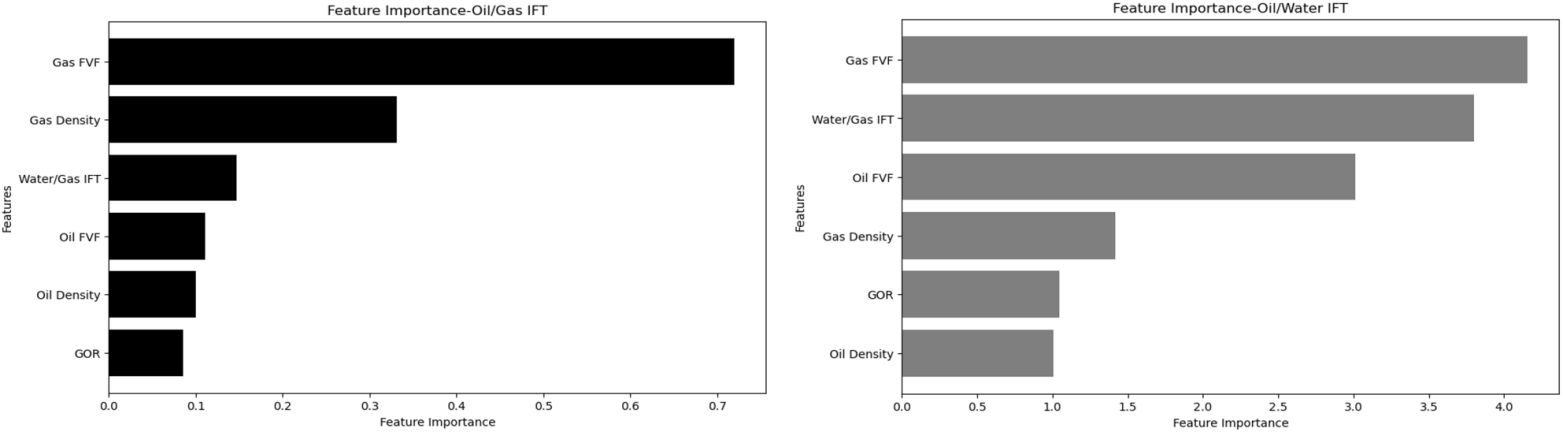
The feature importance of SVR model for both oil/gas IFT and oil/water IFT.

Machine learning methods are often perceived as “black boxes” due to their complex relationship between input parameters and output. Consequently, there is an increasing interest in explainable machine learning techniques. One such approach is the analysis of parameter importance, which helps identify the most influential input parameters affecting the model’s output. Hence, Figs. 7 and 8 present the feature importance analysis of SVR and Catboost models, serving as representatives for the other models, for both Oil/Gas IFT and Oil/Water IFT scenarios.

As can be seen from Fig. 7, Gas FVF is the most important feature for Oil/Gas IFT prediction using Catboost model. This is consistent with the fact that Gas FVF is a measure of the volume occupied by a unit mass of gas at reservoir conditions, which affects the density and pressure of the gas phase. Oil Density is the most important feature for Oil/Water IFT prediction using Catboost model. This is in agreement with the observation that Oil Density reflects the composition and molecular weight of the oil phase, which influences the solubility and adsorption of surfactants at the interface^32^. As can be seen from Fig. 8, Gas FVF is also the most important feature for both Oil/Gas IFT and Oil/Water IFT prediction using SVR model. This suggests that Gas FVF has a strong correlation with both types of IFT, regardless of the machine learning model used.

Some statistical indices were also reported in Tables 6 and 7 for further analysis of the models. These tables show the performance of the proposed models for prediction of Oil/Gas IFT and Oil/Water IFT using different metrics, such as root mean square error (RMSE), mean absolute error (MAE), and coefficient of determination (R^2^).

**Table 6:** Statistical indices used for describing the performance of proposed models for oil/gas IFT.

Model	Train			Test			Overall		
	RMSE	MAE	R^2^	RMSE	MAE	R^2^	RMSE	MAE	R^2^
Gradient boosting	0.097	0.009	0.998	0.123	0.032	0.997	0.406	0.020	0.997
XGBoost	0.021	0.009	0.999	0.091	0.036	0.998	0.056	0.022	0.998
CatBoost	0.045	0.015	0.999	0.069	0.025	0.999	0.057	0.020	0.999
Decision tree	0.138	0.057	0.996	0.197	0.081	0.992	0.167	0.069	0.994
Random forest	0.147	0.084	0.997	0.395	0.228	0.980	0.271	0156	0.988
Support vector regressor	0.012	0.008	0.999	0.014	0.009	0.999	0.013	0.008	0.999

**Table 7:** Statistical indices used for describing the performance of proposed models for oil/water IFT.

Model	Train			Test			Overall		
	RMSE	MAE	R^2^	RMSE	MAE	R^2^	RMSE	MAE	R^2^
Gradient boosting	0.657	0.153	0.947	0.723	0.276	0.938	0.690	0.214	0.941
XGBoost	0.257	0.197	0.992	0.429	0.330	0.977	0.343	0.263	0.984
CatBoost	0.249	0.162	0.992	0.328	0.217	0.986	0.288	0.189	0.989
Decision tree	0.895	0.693	0.903	1.006	0.786	0.873	0.950	0.739	0.888
Random forest	0.147	0.084	0.997	0.395	0.228	0.980	0.271	0.156	0.988
Support vector regressor	0.049	0.033	0.999	0.051	0.036	0.999	0.500	0.034	0.999

Approximately all implemented models show promising results in both cases; Oil/Gas IFT and Oil/Water IFT. The exceptional performance of the XGBoost model can be attributed to significant advancements in the Gradient Boosting Decision Tree (GBDT) technique, leading to improvements in three key aspects. Firstly, XGBoost surpasses traditional GBDT by utilizing second-order Taylor expansion, incorporating enhanced residuals at both the first and second orders. This enhancement expands the applicability of the XGBoost model across a broader range of applications. Secondly, XGBoost incorporates a regularization term into its objective function, effectively managing the model’s complexity. By doing so, it reduces variance and mitigates the risk of overfitting during the training process. Furthermore, XGBoost employs the random forest column sampling method, which further enhances its robustness by reducing the probability of overfitting.

The XGBoost model has demonstrated outstanding learning performance and training speed. To demonstrate the model’s robustness, we performed a five fold cross-validation on the training dataset. fivefold cross-validation is well-suited for the Interfacial Tension dataset as it offers a compromise between speed and predictive precision. By cycling through five unique training and testing groups, it yields a dependable performance measure, avoids the inconsistencies of a one-off split, and counteracts overfitting, thus enhancing the model’s adaptability to unseen data. In this approach, the training set is divided into k subsets, and the model is trained using k-1 folds while being validated on the remaining portion of the data. The performance measure reported by the k-fold cross-validation is the average of the values computed for each fold, providing a comprehensive assessment of the model’s performance.

**Figure 9:**
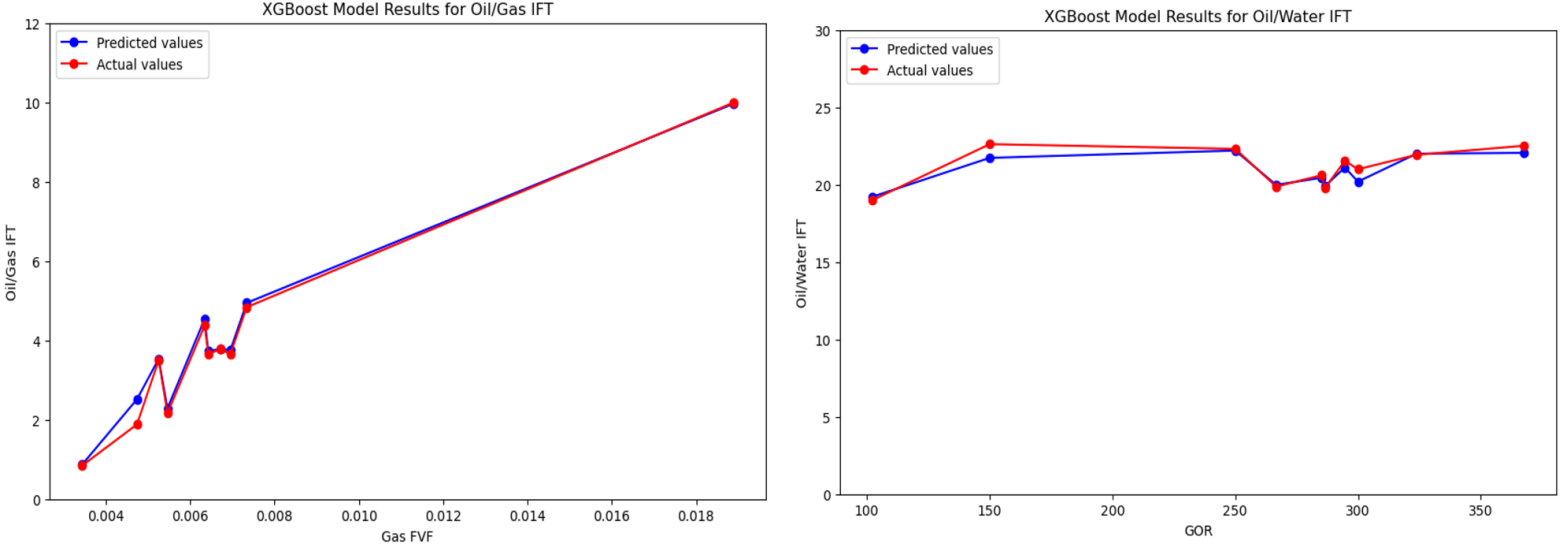
Graphical illustration for comparison between experimental oil/gas IFT and oil/water values and the XGBoost model estimations at various GOR and gas FVF.

In order to fully discover the ability of implemented models to predict the outputs, we used the XGBoost model to show the comparison between experimental Oil/Gas IFT and Oil/Water IFT values and the XGBoost model estimations at various GOR and Gas FVF in Table 8. Also, in order to provide a better outlook a graphical illustration is presented in Fig. 9 corresponding to Input and output data.

**Table 8:** Samples used for comparison between experimental oil/gas IFT and oil/water IFT values and the XGBoost model estimations at various GOR and gas FVF.

Gas FVF	Actual value	Predicted value	GOR	Actual value	Predicted value
Oil/gas IFT			Oil/water IFT		
0.018883	9.9900	9.9643	102.303	19.0030	19.2218
0.003435	0.8424	0.8687	150	22.6175	21.7317
0.004737	1.8823	2.5157	250	22.3158	22.2052
0.006953	3.6570	3.7559	266.666	19.8789	19.9845
0.006428	3.6677	3.7227	284.985	20.6014	20.4448
0.006721	3.7723	3.7874	286.626	19.8027	19.9168
0.007340	4.8358	4.9439	294.614	21.5364	21.1050
0.005460	2.1687	2.2874	300	20.9990	20.1986
0.006341	4.3697	4.5393	323.834	21.9288	22.0028
0.005252	3.5027	3.5347	367.463	22.0515	22.0568

The XGBoost model is based on the GBDT technique, in which the boosting strategy is adopted to integrate several decision trees through a powerful and efficient technique. The number of trees depends on the number and type of data; hence, a strong learner is created. However, the DTs model is among the machine learning approaches that employ a tree-like framework to handle a wide range of input types and find the appropriate path for the prediction of results. At the same time, the DTs model can sometimes be vulnerable to overfitting. It is also sensitive to the noise in data. The concurrent use and integration of several DTs models can compensate for the lack of accuracy in each model and reduce the overall error. As a result of this procedure, the models like XGBoost that have been developed through the GBDT can outperform the DTs models in estimating the outputs.

### Advantages

- A wide range of models: our use of a comprehensive set of machine learning models provides a robust analysis of different algorithms’ capabilities in predicting interfacial tension (IFT) within oil and gas systems.
- Rigorous performance evaluation: the application of multiple performance metrics (R-squared, MAE, RMSE) allows for a nuanced assessment of each model’s predictive accuracy.
- Feature importance analysis: by conducting a feature importance analysis, we have contributed to a deeper understanding of the critical parameters influencing IFT, which can inform the optimization of the processes in the industry.
- Practical implications: through empirical evaluation, our study offers valuable insights for reservoir management strategies, potentially assisting in the development of more efficient methods within the sector.

### Limitations

Our study offers valuable insights into predicting oil/gas IFT and oil/water IFT using several machine learning algorithms. However, there are some limitations that should be noted and addressed in future research:

- Data dependence: the performance of machine learning models depends largely on the quality of the data used to train and test them. In our study, we used a dataset that was collected retrospectively, which may introduce some biases or errors that could affect the accuracy of our predictions. Therefore, it is important to validate our models using more reliable and diverse data sources.
- Model assumptions: machine learning algorithms assume that the patterns they learn from the training data can be generalized to new and unseen data. However, this assumption may not hold if the data distribution changes over time (a phenomenon known as concept drift). This may cause the performance of the model to degrade over time. Therefore, it is essential to monitor and update our models periodically to account for possible changes in the data.
- Model interpretability: some of the machine learning algorithms used in our study, such as XGBoost and CatBoost, are complex and difficult to interpret. This may limit their practical usefulness in some situations where explainability and transparency are required. Therefore, it is advisable to use simpler and more interpretable models, such as decision trees and gradient boosting, when possible, or to employ techniques such as feature importance analysis or SHAP values to enhance the interpretability of complex models.
- Computational complexity: as noted by the review, different models require different computational efforts, which can be a limiting factor when deploying these models in real-time or on low-resourced computational systems.
- Dynamic changes in systems: the static nature of machine learning predictions does not account for real-time changes in system parameters, thereby limiting the use in dynamic settings without retraining the model with new data.

## Conclusions

This article compared the performance of six machine learning models, namely SVR, RF, DT, GB, CB, and XGB, in predicting IFT between oil/gas and oil/water systems. The main results are summarized as follows:

- In this study, we consider a wide range of input parameters that affect the IFT of both oil/gas and oil/water systems, unlike most of the previous studies that have neglected some of these parameters or focused on specific cases.
- Unlike most of the previous studies that have concentrated on one type of fluid pair, in this study, we investigate two types of output parameters, namely oil/gas IFT and oil/water IFT.
- Support Vector Regression (SVR) and Catboost (CB) performed best for oil/gas and oil/water IFT prediction.
- Random Forests (RF) exhibited lower accuracy across both scenarios.
- XGBoost (XGB) demonstrated excellent robustness and training speed due to its advanced techniques.
- All models showed over 90% variability prediction in IFT, indicating high performance.
- For oil/gas IFT, SVR had the lowest MAE (0.009) and RMSE (0.014); RF had the highest.
- For oil/water IFT, SVR also had the lowest MAE (0.036) and RMSE (0.051), outperforming CB’s 0.986 accuracy.

## Data Availability

The datasets generated and/or analyzed during the current study are not publicly available due to confidentiality agreements with the authors and the data providers. The data are available from the corresponding author on reasonable request and subject to approval from the relevant parties.
